# The validation of a questionnaire to assess barriers to enteral feeding in critically ill patients: a multicenter international survey

**DOI:** 10.1186/1472-6963-14-197

**Published:** 2014-05-01

**Authors:** Naomi E Cahill, Lauren Murch, Miao Wang, Andrew G Day, Deborah Cook, Daren K Heyland

**Affiliations:** 1 Department of Public Health Sciences, Queen’s University, 99 University Ave, Kingston, ON K7L 3N6, Canada; 2 Clinical Evaluation Research Unit, Kingston General Hospital, 76 Stuart Street, Kingston, ON K7L 2 V7, Canada; 3 Departments of Medicine, Clinical Epidemiology & Biostatistics, McMaster University, 1280 Main St W, Hamilton, ON L8S 4L8, Canada; 4 Department of Medicine, Queen’s University, 99 University Ave, Kingston, ON K7L 3N6, Canada

**Keywords:** Barriers, Critical care, Enteral nutrition, Instrument development, Nutrition therapy, Quality improvement, Multi-level regression analysis, Validity

## Abstract

**Background:**

A growing body of literature supports the need to identify and address barriers to knowledge use as a strategy to improve care delivery. To this end, we developed a questionnaire to assess barriers to enterally feeding critically ill adult patients, and sought to gain evidence to support the construct validity of this instrument by testing the hypothesis that barriers identified by the questionnaire are inversely associated with nutrition performance.

**Methods:**

We conducted a multilevel multivariable regression analysis of data from an observational study in 55 Intensive Care Units (ICUs) from 5 geographic regions. Data on nutrition practices were abstracted from 1153 patient charts, and 1439 critical care nurses completed the ‘Barriers to Enterally Feeding critically Ill Patients’ questionnaire. Our primary outcome was adequacy of calories from enteral nutrition (proportion of prescribed calories received enterally) and our primary predictor of interest was a barrier score derived from ratings of importance of items in the questionnaire.

**Results:**

The mean adequacy of calories from enteral nutrition was 48 (Standard Deviation (SD)17)%. Evaluation for confounding identified patient type, proportion of nurse respondents working in the ICU greater than 5 years, and geographic region as important covariates. In a regression model adjusting for these covariates plus evaluable nutrition days and APACHE II score, we observed that a 10 point increase in overall barrier score is associated with a 3.5 (Standard Error (SE)1.3)% decrease in enteral nutrition adequacy (p-values <0.01).

**Conclusion:**

Our results provide evidence to support our *a priori* hypothesis that barriers negatively impact the provision of nutrition in ICUs, suggesting that our recently developed questionnaire may be a promising tool to identify these important factors, and guide the selection of interventions to optimize nutrition practice. Further research is required to illuminate if and how the type of barrier, profession of the provider, and geographic location of the hospital may influence this association.

## Background

In many areas of healthcare there is a gap between what research evidence indicate ought to be done and what actually happens in clinical practice [1]. The recognition of this problem, together with a heightened focus on quality improvement and evidence-informed practice has stimulated interest in research examining the causes of this ‘knowledge-practice gap’ [2]. A growing body of literature supports the need to identify factors that limit or restrict implementation of best practices [2-6], so that interventions can be selected to address these barriers and improve care delivery.

For instance, when we consider nutrition therapy in critically ill patients, on the one hand, several Clinical Practice Guidelines (CPGs) have been published summarizing evidence from over 200 randomized controlled trials (RCTs) [7-12]; while on the other, observational studies of nutrition practice consistently report large variation in practices across Intensive Care Units (ICUs) [13-16]. Overall, the provision of nutrition therapy is suboptimal, with patients, on average receiving less than 60% of their prescribed calories and protein [13].

To gain a better understanding of the reasons for this knowledge-practice gap in critical care nutrition, we conducted multiple case studies in 4 ICUs in Canada [17]. This qualitative analysis was guided by one of the most often cited theoretical frameworks regarding barriers to knowledge use, the knowledge-attitudes-behaviour framework, by Cabana *et al.*[6]. The analysis led to the development of an extended and revised framework which provided a comprehensive description of factors impeding adherence to critical care nutrition guidelines [18]. Although useful in illuminating potential barriers, this framework did not enable the identification and measurement of these barriers. The ability to assess and quantify barriers is necessary to be successful at tailoring interventions to overcome them and improve practice [5]. Consequently, we developed the ‘Barriers to Enterally Feeding Critically Ill Patients’ Questionnaire [19]. We focused on the provision of enteral nutrition (EN) (i.e. nutrition delivered via a tube placed into the gastrointestinal tract), rather than other aspects of nutrition therapy such as parenteral nutrition (PN) (i.e. intravenous feeding) or nutrient supplementation, because it is the preferred type of feeding [13], practice recommendations related to EN are uniformly endorsed across published guidelines [7-11], and critical care providers generally agree with the recommendations [20].

If this questionnaire is to be a useful tool in identifying barriers to target for change, we need some evidence that the perceived barriers identified by critical care providers completing the questionnaire actually impede the provision of nutrition in the ICU. Thus the purpose of the present study is to gain evidence to support the construct validity of our developed questionnaire by testing the hypothesis that provision of nutrition is lower in ICUs that report the presence of important barriers. As items in the questionnaire focused on the provision of EN, *a priori* we surmised that while we expected to observe an inverse association between barriers to feeding critically ill patients and the amount of total prescribed calories received, the association would be stronger with prescribed calories received from EN.

## Methods

### Study design

The data were collected as part of the International Nutrition Survey, an ongoing global quality improvement initiative in critical care nutrition [21]. This initiative, launched in January 2007, aims to describe and compare nutrition practices in ICUs across the world, enabling the identification of gaps between current nutrition practice and the recommendations of CPGs; a second component is monitoring of change in practices over time. ICUs are invited to participate through mail-outs to membership lists of critical care and nutrition associations from around the world, and advertisements at various international conferences and on websites including our own research groups: http://www.criticalcarenutrition.com. To be eligible, ICUs must have a minimum of 8 beds and have an individual with adequate knowledge of clinical nutrition to be able to complete the data collection (e.g., registered dietician). The initiative involves a bi-annual audit of nutrition practice. To date, there have been 5 survey cycles involving more than 150 ICUs in each year. The most recent survey commenced in May 2013. As part of the 2011 cycle, ICUs were also invited to distribute the ‘Barriers to Enterally Feeding Critically ill Patients’ questionnaire to their ICU staff. As there is no remuneration for participating in the International Nutrition Survey, we provided an incentive of a travel bursary to a scientific meeting to the individuals responsible for co-ordinating data collection at sites who completed both the nutrition audit and the barriers questionnaire.

### Data collection: nutrition audit

Participating ICUs identified a minimum of 20 consecutive adult patients who were mechanically ventilated within the first 48 hours of admission to ICU and who remained in ICU for more than 72 hours. Data were retrospectively abstracted from the patients’ hospital records on their sex, age, admission category (surgery vs. medical), APACHE II score and diagnosis category, height, weight, and baseline nutrition assessment (i.e. energy and protein prescribed by the dietician). Daily nutrition information was collected on the type (i.e. EN, PN, oral, none) and amount of nutrition received (total calories and protein received from EN or PN) from ICU admission for a maximum of 12 days unless death or ICU discharge occurred sooner. Data was not collected on the amount of oral nutrition received during the observation period. Patients were followed while in hospital and their ICU and hospital outcomes determined at 60 days. Abstracted data were entered online using a secure web-based data collection tool (REDCap Software, Version 3.3.0, © 2012 Vanderbilt University).

### Data collection: barriers questionnaire

Development of the questionnaire was guided by our conceptual framework [18], literature review, and existing barriers questionnaires developed for use in other settings [22-25]. As critical care nurses are the primary providers implementing the nutrition plan of care for patients at the bed-side, the questionnaire was intended to be administered to nurses to identify modifiable barriers (i.e., factors amenable to change through a tailored intervention) to enterally feeding critically ill patients. Pilot testing of the questionnaire established content and face validity, and acceptable internal reliability. Exploratory factor analysis indicated an orthogonal 5-factor solution that accounted for 72% of the variance in barriers. We labeled the factors: 1) guideline recommendations and implementation strategies, 2) ICU resources, 3) dietician support, 4) delivery of EN to the patient, and 5) attitudes and behaviour of critical care provider. Details of the development and preliminary validation of the questionnaire have been reported elsewhere [19].

The developed questionnaire is composed of 2 sections. The first section lists 26 potential barriers to delivery of EN and asks the respondent to rate their importance as barriers in their ICU on a 7-point likert scale. These 26 items are divided into 5 subscales corresponding to the 5 factors. Part B includes 6 questions about the personal demographics of the respondent. Table 1 provides an overview of the content of the questionnaire.

**Table 1:** Summary of the barriers to enterally feeding critically Ill patient questionnaire

**Questionnaire section**	**Rationale**	**Number of items**	**Example item**
Part A: Barriers to Delivery of Enteral Nutrition^*^			
Subscale 1: Guideline Recommendations and Implementation Strategies	The characteristics of the guidelines themselves and the methods selected to implement them can impede their application (e.g. wording, level of supporting evidence, format)	6	The current national guidelines for nutrition are not readily accessible when I want to refer to them.
Subscale 2: ICU Resources	Resource constraints hinder staffs ability to adhere to recommendations	3	Enteral formula not available on the unit.
Subscale 3: Dietician Support	As the provider most responsible for nutrition, lack of dietician support can impede the provision of adequate nutrition	4	No or not enough dietician coverage during evenings, weekends, and holidays
Subscale 4: Delivery of Enteral Nutrition to the Patient	Guideline adherence may be more difficult in complex patients	7	In resuscitated, hemodynamically stable patients, other aspects of patient care still take priority over nutrition.
Subscale 5: Critical Care Provider Attitudes and Behaviour	Inadequate knowledge of or negative attitudes towards nutrition guidelines may translate into the behaviour of not adhering to guideline recommendations	6	Fear of adverse events due to aggressively feeding patients
Part B: Personal Characteristics of Respondent	-	6	-

^b^* = 1 = Not at all important, 2 = Unimportant, 3 = Somewhat unimportant, 4 = Neither important or unimportant, 5 = Somewhat important, 6 = Important, and 7 = Very important.

Barriers to Enterally Feeding Critically Ill Patients Questionnaire is available online at http://www.criticalcarenutrition.com.

At the same time as the nutrition audit, the barriers questionnaire was administered to all full and part-time nurses working in participating ICUs. If the nursing pool exceeded 85, a random sample of 60 nurses was used. The questionnaire was distributed according to a modified Dillman’s tailored design method [26], including a pre-contact memo and multiple reminders. The modes of distribution and methods of capturing responses were determined by the dietician or provider responsible for the study locally. The questionnaires were either e-mailed, hand delivered, or placed in staff mailboxes. Questionnaires could be completed online (SurveyMonkey®, Palto Alto, California) or on paper. Paper-based questionnaires were returned to a box placed in the ICU and entered online by the local investigator. Questionnaires responses entered online automatically populated a database.

### Primary outcome: adequacy of calories from enteral nutrition

The primary outcome was defined as the average daily calories received from EN during the first 12 ICU days expressed as a percentage of the baseline caloric prescription. Patients with a contraindication to receiving EN (i.e. mechanical bowel obstruction, bowel ischemia, small bowel ileus, small bowel fistulae, gastrointestinal perforation, and short gut syndrome) were excluded from the analysis. Days without EN including days with exclusive PN were counted as 0% adequacy. Days following permanent progression to exclusive oral intake were excluded from the calculation of EN adequacy.

### Secondary outcome: adequacy of total nutrition

Adequacy of total nutrition included calories from PN and propofol in addition to EN and did not exclude patients with a contradiction to EN but was otherwise calculated the same as the primary outcome.

### Primary predictor: overall barrier score

Individual nurses’ responses to the barriers questionnaire were averaged to the ICU level. Each item was awarded 1, 2, or 3 points if the respondent identified it as a 5 = ‘somewhat important’, 6 = ‘important’ or 7 = ‘very important’ barrier respectively. If an item was rated 1–4 (i.e. ‘not at all important’ to ‘neither important or unimportant’ a 0 score was awarded. The scores of each individual item included in a given subscale was divided by the maximum potential score (i.e. 3) and multiplied by 100, giving a potential range for the barrier score of 0 to 100. The mean score for all 26 items was then calculated to obtain an overall barrier score for each site. We selected to evaluate a 10 point change in barriers score because in the recent pretest posttest feasibility study of tailored guideline implementation strategies (The PERFECTIS Study) [27,28], we observed a 10 point change in barriers score across the 5 participating sites following the intervention. Consequently, we inferred that a 10-point change is clinically achievable.

To explore if the association between barriers and nutrition differed by the type of barrier, we also ran models with the mean barriers score for each of the 5 subscales as the primary predictor of interest. In addition, we were concerned that the mean site level barrier score might be a biased estimate of the true site average if only a few questionnaires were completed at a site, therefore we conducted a sensitivity analysis by running models excluding ICUs with less than 10 completed barrier questionnaires.

### Covariates: ICU and patient

ICU level covariates considered in the analysis included: geographic region, hospital type (i.e., teaching vs. non-teaching), ICU type (open (i.e., patient under the care of any attending physician) vs. closed (i.e., patient under the care of an intensivist), hospital size, ICU size, proportion of nurse respondents working in the ICU for greater than 5 years and proportion of nurse respondents working in a leadership role.

Patient level covariates included: type of admission (surgical vs. medical), admission diagnosis, sex, age, Body Mass Index (BMI), and Acute Physiology and Chronic Evaluation (APACHE) II score (i.e., measure of severity of illness).

### Statistical analysis

ICU and patient level variables were summarized using standard descriptive statistics. The two level hierarchical data with patients (i.e., level I) nested within ICUs (i.e., level II) were analyzed using a mixed effects model with random intercepts to account for site clustering. As provider level data were not associated with specific patients, provider level data (including barriers score) were averaged to the site level and treated as site-level variables. Statistical analysis was completed using PROC MIXED in SAS v9.1.3 (SAS Institute Inc., Cary, NC, USA).

### Assessment of effect modification

*A priori* we hypothesized that the association between barriers score and adequacy of EN may differ within different levels of hospital type, ICU type, and admission category. We assessed potential effect modification by including an interaction term between barriers score and the potential effect modifier in the primary predictor-outcome models. A p-value of <0.10 for interaction terms was considered significant. If no significant interaction was observed we proceeded to include these variables in our assessment of confounding.

### Selection of potential confounders

All analyses were adjusted for evaluable nutrition days and APACHE II score. As nutrition is often started gradually with little received in the first few days of ICU stay, we needed to account for the confounding effect of length of time in the ICU on nutrition adequacy (i.e., patients with short length of stays have lower adequacy than patients will longer length of stay). In addition, as it is difficult to provide adequate nutrition to sicker patients, *a priori* we aimed to account for the effect of severity of illness by including APACHE II scores in all models.

To reduce the number of variables to be evaluated as potential confounders, we first examined the association between the primary outcome and each individual covariate. A p-value of <0.25 in these single predictor models was used to identify covariates for further evaluation [29]. Confounders were selected for inclusion in the adjusted models using the change in estimate method, with a 10% change considered important [30].

### Sample size

With 55 participating centres, we obtain about 80% power at a two-sided alpha = 0.05 if the partial correlation after controlling for covariates between the site average in the barrier scores and the site average in nutritional adequacy was 0.36 (i.e. R-squared = 13%). Thus, we have adequate power to detect moderate to large correlations between nutritional adequacy and site averaged barrier scores. However, the study had limited power for the assessment of effect modification (interaction), which was considered a secondary exploratory study aim.

Institutional ethics approval was obtained from the Queen’s University Health Sciences and Affiliated Teaching Hospitals Research Ethics Board, Kingston, Ontario, Canada, for the conduct of the International Nutrition Survey and at additional centers if required for their participation. The need for informed patient and provider consent was waived given the observational nature and de-identified data capture of this study.

## Results

**Figure 1:**
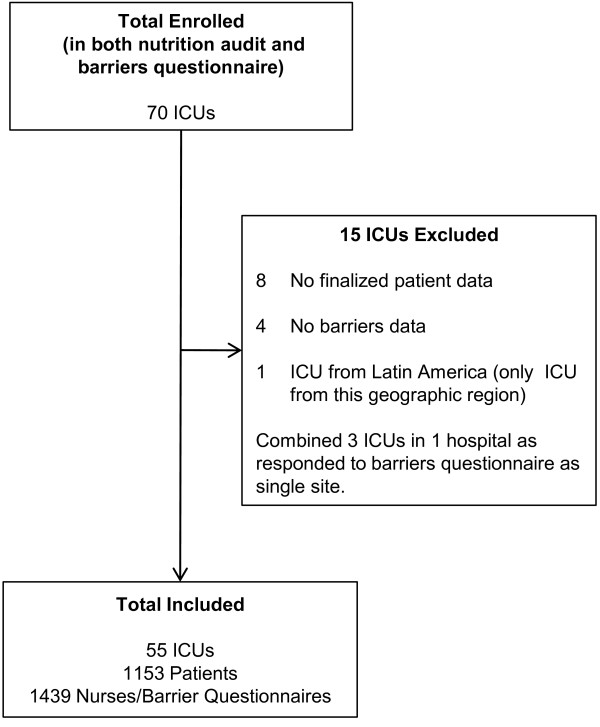
**Flow diagram of study sample.** ICU = Intensive Care Unit.

In total, 55 ICUs were included in the analysis, and 1153 patients were accrued across these sites. Figure 1 shows how the study sample was determined. Tables 2 and 3 report the ICU and patient characteristics. The majority of ICUs were closed units (78%) in teaching hospitals (75%) located in Australia and New Zealand (40%) or North America (33%). Included patients had a mean age of 61 years (standard deviation (SD)) 17, were predominantly admitted with a medical condition (65%) and a mean APACHE II score of 22 (SD 8). Twenty three percent died within 60 days of their ICU admission.

**Table 2:** Characteristics of participating intensive care units (n = 55)

**Intensive care unit characteristic**	**N**	**%**
**Region**		
Canada	7	12.7
Australia and New Zealand	22	40.0
USA	11	20.0
Europe	8	14.5
Asia	7	12.7
**Hospital type**		
Non-teaching	14	25.5
Teaching	41	74.5
**ICU type**		
Other	1	1.8
Closed	43	78.2
Open	11	20.0
Case mix*		
Medical	48	87.3
Neurological	31	56.4
Surgical	51	92.7
Neurosurgical	25	45.5
Trauma	28	50.9
Cardiac surgery	10	18.2
Pediatrics	5	9.1
Burns	10	18.2
	**Mean**	**SD**
**Size of hospital (Beds)**	535	313
		
**Size of ICU**	18	11
		
**% Questionnaire respondents worked in the ICU > 5 years**	55	24
		
**% Questionnaire respondents with leadership role**	36	19

*Type of underlying diseases/conditions treated in the ICU (e.g. a ‘mixed ICU’ may indicate that they treat all the categories, while a ‘Burns unit’ would select ‘burns’ only). Percent column adds up to greater than 100%.

**Table 3:** Personal characteristics and clinical outcomes of patients (n = 1153)

**Patient characteristics**	**N**	**%**
Sex		
Male	687	59.6
Female	466	40.4
Type of admission		
Medical	748	64.9
Surgical elective	119	10.3
Surgical Emergency	286	24.8
Admission diagnosis		
Cardiovascular/vascular*	143	12.4
Respiratory*	294	25.5
Gastrointestinal*	204	17.7
Neurologic*	146	12.7
Sepsis	122	10.6
Trauma*	114	9.9
Other	143	12.4
Contraindication to enteral nutrition		
No	1074	93.1
Yes	79	6.9
Reasons enteral nutrition contraindicated		
* Mechanical bowel obstruction*	11	13.9
* Bowel ischemia*	13	16.5
* Small bowel ileus*	18	22.8
* Small bowel fistulae*	1	1.3
* Gastrointestinal perforation*	33	41.8
* Short gut syndrome*	3	3.8
	**Mean**	**SD**
Age (years)	61	17
Apache II score	22	8
Body mass index	27.5	8
**Clinical outcomes at 60 days**	**Median**	**IQR**
Length of ICU stay (days)^#^	8.8	5.7-15.9
Length of hospital stay (days)^#^	18.9	10.6-35.6
Length of mechanical ventilation (days)^#^	5.8	2.9-12.5
	**N**	**%**
Patient died within 60 days of ICU admission	259	22.5

*includes operative and non-operative admission diagnoses^*#*^*Restrict to 60-day survivorsx*.

**Figure 2:**
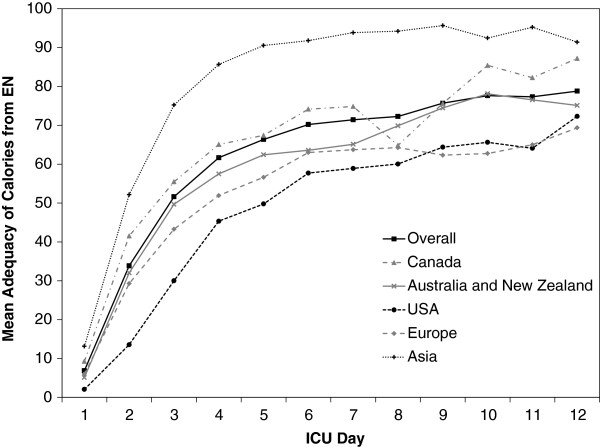
**Mean adequacy of calories from enteral nutrition overall and by geographic region across the 12 days of observation.** EN = Enteral Nutrition.

The majority of patients received EN either alone (n = 819 (71%)) or in combination with PN (n = 189 (16.4%)). Sixty-four patients (5.6%) received PN alone and 81 patients did not receive any artificial nutrition therapy. The mean adequacy of calories from EN and total nutrition were 48% (SD 17) and 60% (SD 16) respectively (Table 4). Figure 2 illustrates the adequacy of calories from EN for all sites and by the 5 geographic regions across the 12 days of observation. A total of 1439 completed barriers questionnaires were included in the analysis. On average the response rate was 30% (range 6 to 62%), equating to a mean of 23 completed questionnaires per ICU (site range 1 to 65). The mean overall barrier score was 23 (SD 11). Table 4 describes the overall and subscale barriers scores by geographic region.

**Table 4:** Mean adequacy of calories from enteral and total nutrition and barrier scores overall and by geographic region

	**Asia**	**Australia and New Zealand**	**Canada**	**Europe**	**USA**	**All**
	**Mean (SD)**	**Mean (SD)**	**Mean (SD)**	**Mean (SD)**	**Mean (SD)**	**Mean (SD)**
**N**	7	22	7	8	11	55
**Adequacy of calories**						
Enteral nutrition	72 (11)	44 (13)	55 (7)	47 (22)	35 (13)	48 (17)
Total nutrition	74 (10)	58 (12)	64 (9)	71 (22)	46 (12)	60 (16)
**Barriers scores**						
Overall	29 (17)	26 (11)	19 (5)	21 (14)	18 (5)	23 (11)
Subscale 1: Guidelines	30 (16)	24 (12)	22 (12)	16 (17)	19 (8)	22 (13)
Subscale 2: Resources	32 (18)	18 (12)	13 (11)	20 (23)	15 (11)	19 (15)
Subscale 3: Dietician	30 (17)	26 (12)	23 (7)	27 (15)	16 (6)	24 (12)
Subscale 4: Patients	28 (17)	30 (13)	21 (9)	25 (14)	25 (6)	27 (12)
Subscale 5: Providers	27 (17)	25 (10)	15 (9)	19 (11)	13 (6)	21 (12)

None of the models evaluating potential effect modification were significant at a p-value of <0.1. Table 5 reports the results of the bivariate analysis of the association between the individual covariates and adequacy of EN. Sex, patient admission type, patient admission diagnosis, proportion of nurse respondents working in the ICU for greater than 5 years, and geographic region were significant at p < 0.25 and were selected, together with hospital and ICU type, to be evaluated as potential confounders using the change-in-estimate criterion. Admission diagnosis was highly collinear with admission type, and as the latter contributed less degrees of freedom than the former, admission diagnosis was not considered further. The estimates changed by greater than 10% between the unadjusted and adjusted models for 4 of the evaluated variables, namely; geographic region (236%), hospital type (13%), patient type (17%), and proportion of respondents working in the ICU > 5 years (51%). Consequently all adjusted analyses controlled for evaluable days, APACHE II score, geographic region, hospital type, patient admission type, and proportion of respondents working in the ICU > 5 years.

**Table 5:** Effect of patient and ICU level variables on adequacy of calories from enteral nutrition

		**Single predictor models**	
	**df**	**Estimate (SE)**	**P-value**
**Patient level variables**			
Age (per decade)	1	-0.16 (0.46)	0.73
Female (versus male)	1	4.19 (1.58)	0.008
Surgical admission type (vs Medical)	1	-18.16 (1.79)	<0.0001
Admission diagnosis	6		<0.0001
Trauma*		Referent	
Cardiovascular/Vascular*		0.48 (3.17)	
Gastrointestinal*		-24.75 (3.01)	
Neurologic*		9.22 (3.03)	
Respiratory*		11.93 (2.88)	
Sepsis		3.20 (3.27)	
Other		0.51 (3.18)	
Apache II score	1	-0.12 (0.11)	0.27
BMI	1	-0.09 (0.10)	0.39
**Site level variables**			
Region	6		<0.0001
USA		Referent	
Asia		31.49 (5.78)	
Australia and New Zealand		10.34 (4.54)	
Canada		18.85 (5.86)	
Europe		8.09 (5.67)	
Teaching (versus non-teaching)	1	-3.40 (4.76)	0.47
Hospital Size (per 1000 beds)	1	-0.20 (6.60)	0.98
ICU beds (per 10 beds)	1	0.20 (0.18)	0.28
Open ICU (versus closed/other)	1	1.28 (5.17)	0.80
% Respondents working in ICU >5 years	1	-0.19 (0.08)	0.02
% Respondents in leadership role	1	0.01 (0.11)	0.91

*Includes operative and non-operative patients.

N = 1070 (due to missing data on 4 patients).

Table 6 shows the results of the unadjusted and adjusted regression models of the association between overall and subscale barrier scores and adequacy of enteral and total nutrition. A significant inverse association was observed, indicating that a 10 point increase in overall barrier score has a negative impact on nutrition practice, resulting in a 3.5 (Standard error (SE) 1.3) and 4.9 (SE 1.3)% decrease in adequacy of calories from enteral and total nutrition respectively. Although a significant association was observed for each of the 5 subscale barrier scores and adequacy of total nutrition, the association was not significant for subscales 1 and 2 with the primary outcome of adequacy of calories from EN. The effect size observed in the sensitivity analysis excluding ICUs contributing less than 10 questionnaires (N = 49) was similar (-3.0 (SE1.3)% and -4.9 (SE 1.3)% for enteral and total nutrition adequacy respectively (p-values <0.05).

**Table 6:** Change in adequacy of enteral and total nutrition associated with a 10 point increase in overall and subscale barrier score

	**Unadjusted**^**1**^			**Adjusted**^**2**^		
	**Estimate**	**SE**	**p-value**	**Estimate**	**SE**	**p-value**
**Adequacy of enteral nutrition**						
Overall barriers score	-1.01	1.84	0.58	-3.54	1.31	0.007
Subscale 1: Guidelines	0.02	1.60	0.99	-1.84	1.20	0.13
Subscale 2: Resources	0.90	1.35	0.51	-1.42	1.04	0.17
Subscale 3: Dietician	-0.71	1.72	0.68	-3.49	1.25	0.005
Subscale 4: Patient	-3.48	1.65	0.04	-4.11	1.11	0.0002
Subscale 5: Providers	0.06	1.77	0.97	-3.61	1.38	0.009
**Adequacy of total nutrition**						
Overall barriers score	-2.82	1.73	0.10	-4.86	1.29	0.0003
Subscale 1: Guidelines	-2.08	1.51	0.17	-3.02	1.20	0.01
Subscale 2: Resources	-1.22	1.30	0.35	-3.24	1.00	0.001
Subscale 3: Dietician	-0.71	1.65	0.67	-3.72	1.30	0.004
Subscale 4: Patient	-4.58	1.52	0.0027	-4.90	1.10	<0.0001
Subscale 5: Providers	-1.38	1.69	0.42	-4.83	1.37	0.0004

Adjusted for evaluable days only.

Adjusted for evaluable days, APACHE II score, hospital type, patient admission type,% respondents working > 5 years in the ICU, and region.

## Discussion

In all areas of healthcare there is a growing interest in identifying and addressing barriers to achieving best practices. However, empirical data demonstrating the negative impacts of barriers or the benefit of overcoming them is sparse, partly due to a lack of validated instruments to measure barriers. To this end we developed a questionnaire to identify important barriers to enterally feeding critically ill patients. The present study provides evidence to support the construct validity of this questionnaire by confirming our *a priori* hypothesis that barriers negatively impact the provision of nutrition in ICUs. Our analysis of data from an observational study involving 1153 patients and 1439 critical care nurses from 55 ICUs across 5 geographic regions demonstrated that after adjusting for important confounding factors, that a 10 point increase in overall barriers score derived from the responses to the questionnaire was associated with a 3.5% decrease in the adequacy of calories from EN.

The results of our analysis are corroborated by the results of our recently completed prospective study evaluating the feasibility of a guideline implementation intervention tailored to overcome barriers to feeding critically ill patients (i.e. PERFormance Enhancement of the Canadian nutrition guidelines by a Tailored Implementation Strategy: The PERFECTIS Study) [27,28]. In that study, which also utilized the Barriers to Enterally Feeding Critically Ill Patients questionnaire, we observed a 10 point decrease in overall barriers score and a 5% increase in total nutrition adequacy following implementation of the tailored intervention. This magnitude of change is equivalent to the change in estimate seen in our regression analysis providing further evidence that barriers are an important factor leading to poor adherence to guideline recommendations in clinical practice.

We would expect that the association between nurse reported barriers to enterally feeding patients and our primary outcome of adequacy of calories from EN to be stronger than with our secondary outcome of adequacy of calories from total nutrition (EN + PN + propofol), because the questionnaire focuses on barrier to the provision of EN and not PN. However, in our regression analyses we observed a 3.5% decrease in EN adequacy compared to 5% with total nutrition adequacy. Furthermore, we observed a significant relationship between all 5 subscales of the barriers questionnaire and total nutrition adequacy, but no association between subscales 1 and 2 with EN adequacy. Further study is required to confirm these observations, explore the reasons for them, and conclude if the association with nutrition adequacy differs by the type of barrier. This knowledge may lead to revisions to the barriers questionnaire and inform the design of interventions whereby barriers that have the greatest impact on nutrition adequacy are targeted.

Although the magnitude of our observed association was statistically significant, the clinical significance of a 3 to 5% change in nutrition adequacy is unclear. Given that on average patients in our study were prescribed 1800 Kcals, a 5% decrease in nutrition adequacy would be equivalent to providing 90 less kcals, which in layman’s terms is the same as a single glass of apple juice per day. We have previously demonstrated that an increase of 1000 kcals per day is associated with a 24% decrease in mortality in this patient group [31]. Consequently, when using this questionnaire, interventions need to target much larger changes in barriers score to ensure that the impact on nutrition outcomes is clinically relevant.

The ‘Barriers to Feeding Critically Ill Patients Questionnaire’ focused on modifiable barriers that were amenable to change at the local level; consequently, the small association observed may in part be because of the presence of other barriers hindering the provision of EN that were not part of the scope of the questionnaire such as ‘higher’ level barriers (e.g. type of health care system, clinical education) or other contextual factors (e.g. leadership support, best practice culture). In a previous analysis, we observed that hospital type (i.e. teaching vs. non-teaching), admission category of the patient (i.e. medical vs. surgical) and sex of the patient (i.e. female vs. male) were significant predictors of EN adequacy [32]; demonstrating that specific hospital, ICU, and patient characteristics can positively or negatively influence nutrition provision. Thus the success of interventions targeting local modifiable barriers may be compromised if these other factors that influence nutrition delivery are not considered.

This approach of analyzing local barriers (using a questionnaire) and tailoring solutions to the identified barriers is foundational to customized knowledge translation, although empirical data showing the success of such an approach is modest [4]. Change directed at documented barriers may not change practice fully, in part because, not all barriers may be identified. Furthermore, it is not yet clear whether customized facilitators of practice change, adapted to local barriers, are more effective than generic facilitators of practice change [4].

The geographic region in which the ICU was located was identified as an important confounding factor of the association between barriers and nutrition adequacy in our dataset. The number of ICUs in each region was small (i.e. 7 to 22), negating our ability to conduct subgroup analyses to better understand the nature of the confounding. Further study is required with more ICUs to confirm this observation. However, it is possible that this variable is a composite measure of other variables that may be associated with the presence of barriers and provision of nutrition such as the type of health care system, models of care delivery, staffing ratios, and education.

There are several limitations that should be considered when interpreting our results. First, participating ICUs were not a random sample of sites but rather a voluntary sample; consequently this sampling strategy may have introduced selection bias if participating sites have a greater interest in nutrition or desire to improve practice compared to the target population. Participating ICUs were predominantly closed units in academic centers, which are two factors that have been associated with higher performance [32]. This may limit the generalizability of our findings. There may have also been selection bias associated with the response rate of 30% for the barriers questionnaire if the perceptions of respondents differed from non-responding nurses. Second, the barriers questionnaires was distributed to critical care nurses, responses may differ by profession therefore the observed association needs to be confirmed amongst dieticians and physicians. Third, the barriers questionnaire was distributed at the same time as the chart audit of nutrition practice; however, we cannot be certain that the nurses who completed the questionnaire are the same as those who cared for the patients included in the study. However, respondents were asked to identify important barriers based on their general experience in the ICU and not with regard to a specific patient, therefore this discrepancy should not have biased the results. Fourth, as with any self-administered survey, responses to the barriers questionnaire reflect factors that the nurses’ perceive to be important barriers in their ICU, which may not be synonymous with ‘true’ barriers. Consequently, the averaged site-level responses can only approximate the true ICU average barrier score with measurement error, resulting in regression dilution. This may have attenuated our estimates of the association between nutrition adequacy and the true barriers score at the ICU. Fifth, the nutrition practice data were abstracted from the patients’ hospital chart; therefore the accuracy of these data depends on accurate chart documentation. Sixth, there is considerable controversy in the nutrition literature as to the optimum nutrition requirements during critical illness. In our study, the prescription of goal calories was determined at the local site by the dietician or physician and therefore their clinical judgment may have influenced the primary and secondary outcome of enteral and total nutrition adequacy. Finally, as in any observational study, there may be residual or unmeasured confounding not accounted for by the regression model.

## Conclusion

In a large sample of international ICUs, we observed that barriers to enterally feeding critically ill patients (measured by a recently developed questionnaire) are inversely associated with nutrition adequacy (measured by a chart audit). Our results provide evidence to support the conceptual underpinnings of knowledge translation research that barriers impede adherence to guideline recommendations in clinical practice. Further research is required to evaluate whether the strength of the observed association differs by type of barrier, profession of the critical care provider, or geographic location of the hospital, and if identifying barriers using our questionnaire can inform interventions that optimize nutrition practice.

## Abbreviations

APACHE: Acute physiology and chronic evaluation; BMI: Body mass index; CPGs: Clinical practice guidelines; EN: Enteral nutrition; ICU: Intensive care unit; PN: Parenteral nutrition; RCT: Randomized controlled trial; SD: Standard deviation; SE: Standard error.

## Competing interests

The authors declare that they have no competing interests.

## Authors’ contributions

NC and DKH were responsible for the study conception and design. LM was responsible for co-ordinating data collection. MW performed the data analysis with assistance from NC. AD, DKH, DC and NC provided methodological and statistical expertise and helped to interpret the results. NC was responsible for drafting the manuscript. All authors contributed to and approved the final manuscript.

## Pre-publication history

The pre-publication history for this paper can be accessed here:

http://www.biomedcentral.com/1472-6963/14/197/prepub
